# In Vitro Inhibition of Bacterial Interactions With Acanthamoeba castellanii by a Monoclonal Antibody Against Galactose-Binding Protein

**DOI:** 10.7759/cureus.109693

**Published:** 2026-05-26

**Authors:** Audrey Folsom, Seyoung Yang, Eun-Jung Kim, Suk-Yul Jung

**Affiliations:** 1 Clinical Laboratory Sciences, Arkansas State University, Jonesboro, USA; 2 Biomedical Laboratory Science, Namseoul University, Cheonan, KOR; 3 Biomedical Laboratory Science, Sanggi University, Wonju, KOR; 4 Clinical &amp; Diagnostic Sciences, Oakland University, Rochester, USA

**Keywords:** acanthamoeba castellanii, amoebic keratitis, galactose, galactose-binding protein, lectin, monoclonal antibody

## Abstract

Introduction

*Acanthamoeba castellanii* causes amoebic keratitis (AK), primarily in contact lens wearers, and severe lesions may require corneal transplantation. Direct contact between the amoeba and the cornea during AK induction is important for the pathogenic process of the amoeba. From a clinical perspective, identifying molecular factors that regulate this contact-dependent interaction is essential for understanding disease progression and for developing preventive or therapeutic strategies for AK.

Methods

This study analyzed the effect of treatments with galactose and monoclonal antibodies against the galactose-binding protein (GBP) on the interactions between bacteria and *A. castellanii*. *A. castellanii *trophozoites were pre-incubated with monosaccharides (galactose, mannose, or glucose; 100 mM) or monoclonal antibodies against GBP for one hour, followed by co-incubation with pathogenic *Escherichia coli *O157:H7 (one hour) or non-pathogenic *E. coli *DH5α (four hours). Bacterial association and invasion were quantified using colony-forming unit (CFU)-based assays following cell lysis, and proteolytic activity was evaluated using gelatin zymography. Statistical analysis was performed using Student’s t-test, with P<0.05 considered significant.

Results

The amoeba-bacteria association after the incubation treatment with mannose was 3.4 times lower than with no treatment. However, the association after the galactose treatment was about 0.7 times lower than with no treatment. In particular, incubation with monoclonal antibodies to GBP showed results very similar to those of mannose. *E. coli* O157:H7’s invasion of the amoebae was reduced by about three times as compared to the association reaction. This reduction in invasion suggests that disruption of lectin-mediated binding may limit the ability of pathogenic bacteria to persist within amoebae. The effects of the incubation treatments with monoclonal antibodies against galactose, mannose, and GBP were so similar that they were almost incomparable. *E. coli* DH5α’s association reaction with galactose was slightly lower than that in the monosaccharide-untreated (no treatment) group. However, it was confirmed that monoclonal antibodies against GBP could reduce the amoeba-bacteria association about 3.65 times more than no treatment. The incubation treatment with antibodies to GBP showed an increase in proteolytic enzyme expression to a degree very similar to that of the mannose treatment group.

Conclusion

Taken together, these findings indicate that GBP plays a key role in contact-dependent interactions relevant to amoebic pathogenicity. Targeting GBP-mediated pathways may represent a clinically meaningful approach to attenuating host tissue damage and microbial interactions associated with AK.

## Introduction

*Acanthamoeba castellanii* causes amoebic keratitis (AK), primarily in contact lens wearers, and severe lesions may require corneal transplantation [1-3]. Among the mechanisms causing lesions, the contact-dependent mechanism can destroy corneal cells, and thus cause corneal damage and inflammation [4]. *Acanthamoeba* trophozoites destroy nerve cells by a contact-dependent pathway and uptake of nerve cells via amoebosomes [5]. During AK induction, direct contact between the amoeba and the cornea is critical for the pathogenic process of the amoeba [6,7], and its galactose-binding proteins (GBPs) can interact with host cells through surface carbohydrates, such as mannose and galactose. In studies on contact-dependent mechanisms, the effects of mannose monosaccharides on cytotoxicity, phagocytosis, and interaction with bacteria have been relatively well explained [8,9]. Not only can mannose bind to lectins (like GBP) on the cell wall surface of amoebae, changing signaling events within the amoeba [10], but the above effects can also be inhibited by antibodies against lectin [11]. Immunization of experimental animals with a mannose-binding protein (MBP) was confirmed to have a protective effect against AK [12]. However, since the effect of an incubation treatment with mannose and MBP alone did not show complete inhibition [13], treatment with other monosaccharides would be necessary. In *Naegleria spp.*, another type of free-living amoeba, it has been reported that mannose and galactose are involved in adhesion and cytotoxicity [10]. There is a need to study the contact-dependent mechanism by treatment with galactose, which has been confirmed to be effective in a similar manner as mannose. The primary objective of this study is to explicitly evaluate the functional role of GBP in the contact-dependent interactions between *A. castellanii* and both pathogenic and non-pathogenic bacteria. Specifically, we aim to analyze the inhibitory effects of a monoclonal antibody against GBP and exogenous galactose on bacterial association, invasion, and subsequent protease activity of the amoebae. By comparing these effects with those of mannose and other monosaccharides, this research seeks to determine whether targeting GBP-mediated pathways represents a viable therapeutic strategy for mitigating the pathogenic processes associated with AK.

This article was previously presented as a poster at the 2025 XXth International Meeting on the Biology and Pathogenicity of Free Living Amoebae on November 6, 2025 at Puerto Morelos, Quintana Roo, Mexico.

## Materials and methods

Culture of *A. castellanii* and *E. coli*, and monoclonal antibody to GBP

*A. castellanii *trophozoites (ATCC No. 50492) were grown on PYG medium (proteose peptone 0.75% (w/v), proteose peptone 0.75% (w/v), yeast extract 0.75% (w/v) and glucose 1.5% (w/v)) in a 75T flask at 30 °C [4]. The medium was changed periodically to maintain confluency above 70% on the microscope [14]. Pathogenic *E. coli *O157:H7 (ATCC No. 43895), which causes hemorrhagic colitis, and non-pathogenic *E. coli* DH5α (KCTC No. 22002) were cultured at 37 °C using tryptic soy agar (TSA, MB cell, Korea) medium. The McFaland turbidity assay was used to determine the number of bacterial colonies, and the concentration of the bacterial culture was adjusted to a turbidity of 0.5, so that 0.5 x 10^3^ to 1.5 × 10^6^ colony-forming units (cfu)/ml were cultured [15]. A monoclonal antibody against a GBP hybridoma clone 2AB2, cultured in a 24-well cell culture plate obtained in a previous study, was used, and it was confirmed to be IgM with a kappa chain [4]. To ensure optimal saturation of GBP, the hybridoma clone 2AB2, possessing an antibody titer of 1.100 or higher, was utilized.

Bacterial association and invasion of *E. coli* into *A. castellanii* by the addition of a monosaccharide or an antibody to GBP

In some previous studies, monosaccharides and bacteria were incubated for 30 min to two hours. For more obvious reasons, monosaccharides saturate the entire amoeba trophozoite cell wall, and incubating the amoeba with bacteria allows more bacteria to associate with and invade the amoeba [4]. Similarly to previous studies, in this study, *A. castellanii *trophozoites were treated with monosaccharides at a concentration of 100 mM or with an antibody to GBP for one hour, the supernatant was discarded, and the amoebae remaining at the bottom of the cell culture plate were used for a mixed culture incubation with bacteria. The amoebae’s incubation treatment with *E. coli* O157:H7 was for two hours, but the incubation with non-pathogenic *E. coli* DH5α was for four hours, which was considered sufficient due to its low rate of association and invasion.

To clearly differentiate between bacterial association and invasion, two distinct protocols were employed. For the association assay, *A. castellanii* trophozoites were incubated with 100 mM monosaccharides (galactose, glucose, and mannose), in 24-well cell culture plates, and all supernatants were removed. After washing three times with phosphate-buffered saline (PBS), an *E. coli* suspension (2 x 10^5^ cfu/0.5 ml of PBS) was added to the plate and incubated at 30 °C for two or four hours. Single colonies of bacteria were diluted with 0.85% NaCl using McFarland turbidity assay [15], then washed three times with PBS, and 0.5% sodium dodecyl sulfate (SDS) was added to destroy all *A. castellanii* trophozoites. Bacterial colonies were counted after adding 20 μL to Luria-Bertani (LB) agar plates and incubating them for one day at 37 °C [14]. Bacterial association was calculated as follows: bacterial colonies grown (cfu)/total bacteria (cfu) × 100 = % of bacteria that associated with *A. castellanii *trophozoites. For the invasion assay, after the initial incubation, the extracellular bacteria were killed by treating the wells with gentamicin (100 µg/mL) for one hr. The amoebae were then washed and lysed, ensuring that the resulting CFU counts represented only the invaded bacteria.

Protease analysis of *A. castellanii* trophozoites by the addition of monosaccharide or antibody to GBP, and statistical analysis

A zymography analysis was performed to analyze the protease activity of *A. castellanii *by combining antibodies against monosaccharides or GBP with *A. castellanii* [16]. As mentioned above, to ensure sufficient binding, monosaccharides or antibodies were incubated with *A. castellanii* trophozoites at 30 °C for two hours. The supernatant was removed, washed three times with PBS, disrupted by sonication, and the entire lysate was generated and added to a 12% sodium dodecyl-polyacrylamide gel electrophoresis (SDS-PAGE) with gelatin. Then, 5% Triton-X100 was added to the gel for one hour at room temperature (RT), and the gel was stained with Coomassie Brilliant Blue. All experiments were performed in triplicate (n=3) and repeated three times as independent biological replicates to ensure reproducibility. Data were analyzed using Student two-sample t-test and one-way analysis of variance (ANOVA) between the control and various treatment groups (monosaccharides and antibody). A value of P<0.05 was considered statistically significant.

## Results

*E. coli *O157:H7 association with and invasion into *A. castellanii* by incubation with a monosaccharide and a monoclonal antibody to the GBP

**Figure 1:**
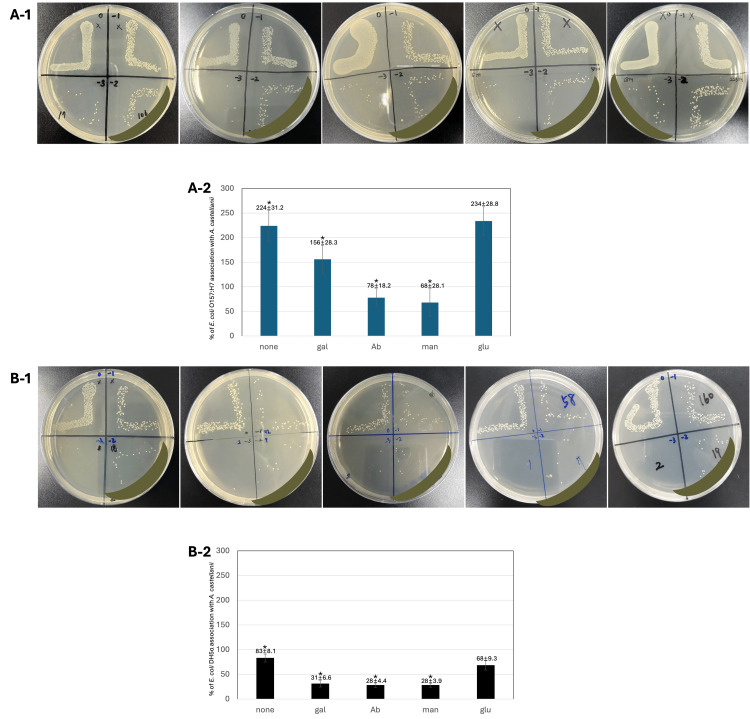
Association of E. coli with A. castellanii trophozoites by the addition of monosaccharides or antibody to GBP A-1 and A-2 indicate *E. coli *O157:H7 association. A-1 shows *E. coli* O157:H7 plates calculated with CFU; A-2 shows a numerical value coming from A-1. Results of B-1 and B-2 were the same as the sequence of A-1 and A-2, but *E. coli *DH5α was used. Statistical analysis was performed using Student’s two-sample t-test (n=3 per group). For A-1 and A-2, compared with the no-treatment group, galactose (t = 2.80, P<0.05), antibody to galactose-binding protein (GBP; t=6.00, P<0.05), and mannose (t=5.26, P<0.05) showed statistically significant differences, whereas glucose did not (t=0.58, P>0.05). For B-1 and B-2, compared with the no-treatment group, galactose (t=8.74, P<0.05), antibody to GBP (t = 10.74, P<0.05), and mannose (t=11.07, P<0.05) showed statistically significant differences, whereas glucose did not (t=2.15, P>0.05). Asterisks indicate statistically significant differences (P<0.05). GBP, galactose-binding protein; Gal, galactose; Ab, a monoclonal antibody to GBP; man, mannose; glu, glucose.

**Figure 2:**
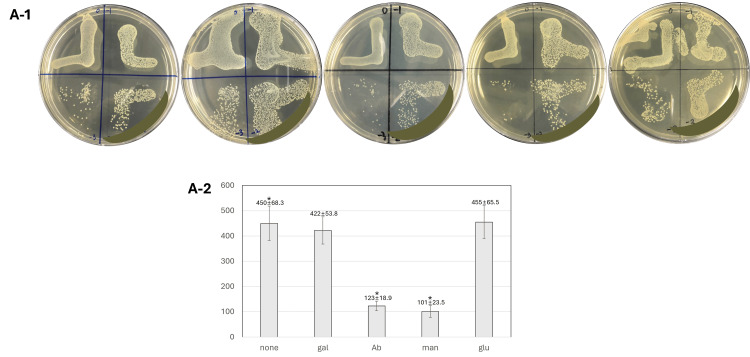
E. coli O157:H7 invasion into A. castellanii trophozoites by the addition of monosaccharides or antibody to GBP A-1 and A-2 indicate *E. coli* O157:H7 invasion. A-1 shows bacterial plates calculated with CFU; A-2 shows a numerical value coming from the A-1. Statistical analysis was performed using Student’s two-sample t-test (n=3 per group). Compared with the none control, galactose showed no significant difference (t=0.55, P=0.61), whereas treatment with a monoclonal antibody to GBP (t=7.85, P<0.05) and mannose (t=8.36, P<0.05) significantly reduced bacterial invasion. Glucose showed no significant effect (t= −0.10, P>0.05). Asterisks indicate statistically significant differences (P<0.05). GBP, galactose-binding protein; Gal, galactose; Ab, a monoclonal antibody to GBP; man, mannose; glu, glucose.

*A. castellanii* trophozoites were incubated with galactose, mannose, or a monoclonal antibody to the GBP for one hour, followed by a mixed culture incubation with *E. coli* O157:H7 for one hour and *E. coli *DH5α for four hours. Afterwards, as explained in the experimental method, the results of culturing the *E. coli* for one day were shown. The protocol to calculate the existing association percentage was to use a mixed culture incubation time of 30 minutes or one hour [17,18]. However, in this study, we specifically attempted to analyze the effects of an incubation treatment with monosaccharides and antibodies by mixing *E. coli *DH5α with *A. castellanii* for a four-hour incubation. As reported in previous studies, mannose had the highest effect on the amoebae. The amoeba-bacteria association with a mannose incubation treatment was 3.4 times ((27x100)/224)) lower than with no treatment (Figures 1A-1, 1A-2).

Meanwhile, the amoeba-bacteria association with a galactose incubation treatment was about 0.7 times lower than with no treatment. In particular, incubation treatment with monoclonal antibodies to GBP showed results very similar to those of mannose. Interestingly, there was a difference in *E. coli* O157:H7 association by incubation treatment with monoclonal antibodies against galactose and GBP. The effect of the monoclonal antibody incubation treatment was to inhibit the association almost twice as much as the incubation with monosaccharides had on bacterial association by binding to the cell wall of *A. castellanii* trophozoites. Taken together, this suggests that bacterial association was inhibited by the treatment with monoclonal antibodies against GBP.

Overall, the *E. coli* O157:H7-trophozoite invasion was reduced by about three times that of the amoeba-bacteria association (Figures 1B-1, 1B-2). In the invasion results, as in the association results, incubation treatment with mannose had the best inhibitory effect. However, when looking only at trophozoite invasion, the inhibitory effects of monoclonal antibodies against galactose, mannose, and GBP were so similar that they were almost incomparable. These results also confirmed that, like association studies, invasion studies could be a good candidate for evaluating the inhibiting effect of incubation treatments with monoclonal antibodies against GBP and galactose.

*E. coli* DH5α association with *A. castellanii* by incubation with a monosaccharide and monoclonal antibody to the GBP

According to previous reports, the amoeba-bacteria association of non-pathogenic *E. coli* DH5α was so low that the association of pathogenic bacteria with the amoebae differed by almost four times that of the non-pathogenic bacteria [19]. Therefore, in this study, the association percentage was calculated by treating non-pathogenic *E. coli *DH5α with *A. castellanii *for a sufficiently longer incubation time, in this case, four hours. Another reason for doing this was that in order to sufficiently analyze the effects of certain factors, such as the incubation treatment with monosaccharides and antibodies, the association percentage in the negative control (the no-treatment group) must be high enough so that the effects of other factors could be compared. The amoeba-*E. coli *DH5α association with the galactose incubation treatment was slightly lower than that in the monosaccharide-untreated group but did not reach statistical significance (Figure 2).

However, it was confirmed that incubation treatment with the monoclonal antibodies against GBP could reduce the association about 3.65 times more than no treatment. In particular, the mannose incubation treatment had the maximum effect, as reported in other research studies [13]. Therefore, the inhibitory effect of the galactose incubation treatment on pathogenicity was present but not high. However, it was observed that the effect of the incubation treatment with monoclonal antibodies to GBP had the greatest inhibitory effect on both pathogenic and non-pathogenic *E. coli*. 

As illustrated in Figures 1, 2, the inhibitory effects of mannose and anti-GBP antibodies were statistically superior to other treatments across all bacterial strains tested. The quantitative reduction in bacterial association and invasion is clearly visualized through the significant divergence in CFU counts (P<0.001, ANOVA), as shown in the bar graphs. Detailed P-values and multiple comparison results for each treatment group are consolidated within the respective figure panels to enhance clarity and reader comprehension.

Proteolytic activities by incubation with a monosaccharide and monoclonal antibody to the GBP

**Figure 3:**
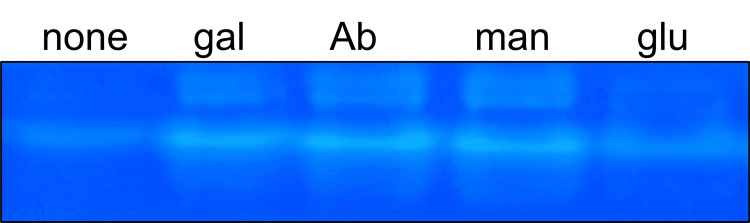
Zymography to A. castellanii by the addition of monosaccharides or antibody to GBP The monosaccharides were treated to *A. castellanii* in 100 mM concentration. GBP, galactose-binding protein; Gal, galactose; Ab, a monoclonal antibody to GBP; man, mannose; glu, glucose.

If monosaccharides bind to lectins, such as GBP, in the *A. castellanii* cell wall, cellular signaling changes can be induced within the *A. castellanii *cytoplasm [10]. Therefore, the expression patterns of the proteases of *A. castellanii *after an incubation treatment with the antibodies and monosaccharides used in this study were observed (Figure 3).

Unlike the untreated group, the expression of proteases in the *A. castellanii* treated with monosaccharides or antibodies was evident. However, the expression of the proteolytic enzymes after an incubation treatment with glucose showed little difference from the untreated group. In the case of the mannose treatment, the expression of the proteolytic enzymes was very distinct and showed an increased pattern as compared to the other groups. Incubation treatment with antibodies to GBP showed an increase in *A. castellanii*’s proteolytic enzymes to a degree very similar to that of the mannose treatment group. However, the effect of the galactose treatment on proteolytic enzyme activity was slightly lower than that of the mannose or antibody incubation treatment.

## Discussion

Although few reports exist on the pathogenicity of free-living amoebae, to which *Acanthamoeba* belongs, interest in pathogenicity- and antigenicity-related proteins remains. For the development of AK, contact-dependent mechanisms such as lectin-mediated, carbohydrate-dependent interactions may trigger downstream contact-independent processes [20], including the induction of proteolytic enzyme expression [21]. Mannose-binding lectin (MBL) binds to mannose residues and can accelerate adhesion, thereby promoting amoebial pathogenicity [22]. Among animal lectins, MBL can play roles such as target cell recognition, signal transduction, and cell adhesion due to its ability to bind to monosaccharides [23,24]. Therefore, MBL can recognize and interact with monosaccharides on the surface of pathogens. This is related to the fact that MBL, that is, mannose-binding protein (MBP) in amoebae, is very important for its interaction with pathogens such as *E. coli*. When mannose, which is involved in the contact-dependent mechanism, is incubated continuously with amoebae, it is confirmed that the vacuoles in the cytoplasm of the amoebae become very large [25]. This means that phagocytosis can become active. On the contrary, if mannose is pre-bound to the amoebae, the interaction with other target cells or bacteria may be poor due to the pre-bound mannose. Therefore, these results will enable us to understand the contact-dependent mechanism in which monosaccharides participate.

From a clinical perspective, these findings may be relevant to contact lens users, who represent the population at highest risk for AK. Disruption of lectin-mediated interactions between *Acanthamoeba* trophozoites and microbial or host surfaces could potentially reduce initial adhesion and subsequent pathogenic processes on the corneal surface. However, given that the present study was conducted under in vitro conditions, direct clinical extrapolation should be made with caution. Furthermore, there is a possibility that MBL could be used for the diagnosis of *Acanthamoeba* [26].

One of the interesting findings in this study was that when non-pathogenic *E. coli *DH5α was mixed-cultured with* A. castellanii* for four hours, very high association percentages were induced, unlike previous studies with shorter incubation times [16]. However, even with this high association, invasion of non-pathogenic *E. coli *DH5α was 0% (data not shown). These results demonstrated that pathogenic *E. coli *O157:H7 and non-pathogenic *E. coli *DH5α have clear differences in their interaction with *A. castellanii* trophozoites.

The activity of proteases induced by a contact-dependent mechanism by incubation treatment with monosaccharides was reported in a study on *A. culbertsoni* [19]. As the mannose concentration in the treatment increased to 100mM, the activity of protease increased, and mannose induced a slightly greater protease expression than galactose. Similar to these study results, the proteolytic activity in *A. castellanii* treated with 100 mM mannose was maximized. Interestingly, the galactose treatment induced a high amount of protease activity, although it was not as strong as mannose, and protease activity was also induced by the treatment with antibodies against GBP.

Importantly, the observation that monoclonal antibodies against GBP reduced amoeba and bacteria association and modulated protease activity suggests that GBP may represent a potential therapeutic target. Targeting GBP-mediated pathways could interfere with early adhesion events and downstream pathogenic signaling. The strength of this study lies in its pioneering use of a specific monoclonal antibody to target and neutralize the GBP of *A. castellanii*, moving beyond simple carbohydrate-inhibition models. By employing both pathogenic (O157:H7) and non-pathogenic (DH5α) bacterial strains, we have provided a comprehensive comparative analysis that highlights the universal importance of GBP in amoebic-bacterial interactions.

This research contributes significantly to the current body of knowledge by identifying GBP as a crucial molecular determinant in the early stages of AK. Furthermore, the demonstrated efficacy of anti-GBP antibodies in reducing bacterial association and invasion offers a novel perspective for developing targeted immunotherapies, shifting the focus from broad-spectrum treatments to specific molecular interventions. The interaction between *A. castellanii* and bacteria is not merely a laboratory phenomenon but serves as a critical proxy for understanding the initial stages of AK in the human cornea.

In the clinical context, the corneal epithelium is often co-colonized by bacteria, especially in contact lens wearers. Our findings suggest that GBP acts as a molecular bridge, where the amoeba utilizes this lectin to recognize and attach to bacterial prey present on the corneal surface. Translating these interactions to human health, the inhibition of GBP, whether by exogenous galactose or specific monoclonal antibodies, could represent a therapeutic intervention. By blocking the GBP-mediated contact, we can theoretically disrupt the amoeba’s ability to establish a stable niche on the cornea, thereby reducing the localized bacterial load that often exacerbates inflammatory tissue destruction. Consequently, targeting these lectin-carbohydrate interactions provides a strategic pathway to mitigate the cascading pathogenic events that lead to severe corneal ulceration and vision loss. 

Nevertheless, these findings are limited to controlled experimental conditions, and further validation using in vivo models or clinical samples is required before considering therapeutic applications. Future studies should focus on the molecular characterization of protease regulation and other antigenic proteins within the cytoplasm of *A. castellanii*, particularly under conditions involving ionic modulation, to further elucidate its pathophysiological mechanisms.

Limitations of the study

While this study primarily focuses on the inhibition of bacterial interactions, it serves as a critical functional validation of GBP as a key mediator in *A. castellanii* pathogenicity. Although the precise downstream signaling pathways activated upon GBP-ligand binding remain to be fully elucidated, our results providing clear evidence of competitive inhibition by both monoclonal antibodies and specific sugars offer a necessary foundation for future mechanistic studies. Identifying these interaction patterns is a prerequisite for targeted molecular research into amoebic signaling complexes. All experiments were conducted under in vitro conditions, which do not fully capture the complexity of host and *A. castellanii* interactions in vivo, particularly within the corneal microenvironment. The mechanisms underlying GBP-mediated inhibition were not fully elucidated at the molecular level. However, the use of a monoclonal antibody targeting GBP represents a particularly important and meaningful aspect of this study, as it provides direct functional evidence for the role of GBP in mediating these interactions. Despite these limitations, the observed inhibitory effects on bacterial association and invasion were robust and consistent, supporting the biological relevance of GBP in mediating *A. castellanii*-bacteria interactions.

## Conclusions

Our data demonstrated that mannose and monoclonal antibodies against GBP significantly reduce the association and invasion of both pathogenic and non-pathogenic bacteria with *A. castellanii* in an in vitro model. These findings, coupled with the observed changes in protease activity, suggest that GBP-related pathways play a substantial role in amoebic pathogenicity. While these results were obtained under controlled laboratory conditions, they provide a valuable mechanistic basis for understanding amoeba and bacterial interactions. Future studies involving in vivo models are necessary to confirm these effects in a complex biological environment; however, our study highlights targeting GBP-mediated pathways as a promising potential strategy for the development of future preventive or therapeutic interventions for AK.

## References

[1] Akbar N, Siddiqui R, El-Gamal MI, Zaraei SO, Saeed BQ, Alawfi BS, Khan NA (2024). Potential anti-amoebic activity of sulfonate- and sulfamate-containing carboxamide derivatives against pathogenic Acanthamoeba castellanii belonging to the genotype T4. Parasitol Int.

[2] Kitzmann AS, Goins KM, Sutphin JE, Wagoner MD (2009). Keratoplasty for treatment of Acanthamoeba keratitis. Ophthalmology.

[3] Siddiqui R, Khan NA (2012). Biology and pathogenesis of Acanthamoeba. Parasit Vectors.

[4] Kim DY, Son DH, Matin A, Jung SY (2021). Production of a monoclonal antibody against a galactose-binding protein of Acanthamoeba castellanii and its cytotoxicity. Parasitol Res.

[5] Pettit DA, Williamson J, Cabral GA, Marciano-Cabral F (1996). In vitro destruction of nerve cell cultures by Acanthamoeba spp.: a transmission and scanning electron microscopy study. J Parasitol.

[6] Cao Z, Jefferson DM, Panjwani N (1998). Role of carbohydrate-mediated adherence in cytopathogenic mechanisms of Acanthamoeba. J Biol Chem.

[7] González-Robles A, Castañón G, Cristóbal-Ramos AR, Lázaro-Haller A, Omaña-Molina M, Bonilla P, Martínez-Palomo A (2006). Acanthamoeba castellanii: structural basis of the cytopathic mechanisms. Exp Parasitol.

[8] Kang AY, Park AY, Shin HJ, Khan NA, Maciver SK, Jung SY (2018). Production of a monoclonal antibody against a mannose-binding protein of Acanthamoeba culbertsoni and its localization. Exp Parasitol.

[9] Sharma C, Khurana S, Bhatia A, Arora A, Gupta A (2023). The gene expression and proteomic profiling of Acanthamoeba isolates. Exp Parasitol.

[10] Jung SY (2023). Association between Naegleria fowleri and bacteria, its cytotoxicity and protein activity by monosaccharides. Acta Microbiol Bulg.

[11] Park AY, Kang AY, Jung SY (2018). Protective effects of a monoclonal antibody to a mannose-binding protein of Acanthamoeba culbertsoni. Biomed Sci Lett.

[12] Garate M, Marchant J, Cubillos I, Cao Z, Khan NA, Panjwani N (2006). In vitro pathogenicity of Acanthamoeba is associated with the expression of the mannose-binding protein. Invest Ophthalmol Vis Sci.

[13] Kim JH, Matin A, Shin HJ (2012). Functional roles of mannose-binding protein in the adhesion, cytotoxicity and phagocytosis of Acanthamoeba castellanii. Exp Parasitol.

[14] Jung SY, Matin A, Kim KS, Khan NA (2007). The capsule plays an important role in Escherichia coli K1 interactions with Acanthamoeba. Int J Parasitol.

[15] Song KJ, Jung SY (2017). Biocidal effects of chlorine dioxide on isolated and identified pathogens from nosocomial environment - biochemical and technical convergence (Article in Korean). J Digit Converg.

[16] Son DH, Kim EJ, Matin A, Jung SY (2022). Interaction between Naegleria fowleri and pathogenic Escherichia coli by mannose and changes in N. fowleri protease. Parasitol Res.

[17] Jung SY (2021). Possibility of saccharide-binding protein to interactions of Acanthamoeba castellanii and several bacteria. Acta Microbiol Bulg.

[18] Jung SY (2021). Effect of mannose to the interactions between Naegleria fowleri and pathogenic bacteria. Biomedicine.

[19] Jung SY (2022). Effect of monosaccharides on the interaction between Acanthamoeba culbertsoni and Shigella sonnei and the cytotoxicity and protease activity of A. culbertsoni. Biomedicine.

[20] Garate M, Cao Z, Bateman E, Panjwani N (2004). Cloning and characterization of a novel mannose-binding protein of Acanthamoeba. J Biol Chem.

[21] Na BK, Kim JC, Song CY (2001). Characterization and pathogenetic role of proteinase from Acanthamoeba castellanii. Microb Pathog.

[22] Carvalho-Kelly LF, Freitas-Mesquita AL, Ferreira Pralon C, de Souza-Maciel E, Meyer-Fernandes JR (2023). Identification and characterization of an ectophosphatase activity involved in Acanthamoeba castellanii adhesion to host cells. Eur J Protistol.

[23] AbuSamra DB, Argüeso P (2018). Lectin-glycan interactions in corneal infection and inflammation. Front Immunol.

[24] Dos Santos Silva PM, de Oliveira WF, Albuquerque PB, Dos Santos Correia MT, Coelho LC (2019). Insights into anti-pathogenic activities of mannose lectins. Int J Biol Macromol.

[25] Yoo KT, Jung SY (2012). Effects of mannose on pathogenesis of Acanthamoeba castellanii. Korean J Parasitol.

[26] Kanakapura Sundararaj B, Goyal M, Samuelson J (2025). Targets for the diagnosis of Acanthamoeba eye infections include four cyst wall proteins and the mannose-binding domain of the trophozoite mannose-binding protein. mSphere.

